# Efficient protection of the Baltic Sea needs a revision of phosphorus metric

**DOI:** 10.1007/s13280-023-01851-2

**Published:** 2023-04-10

**Authors:** Antti Iho, Helena Valve, Petri Ekholm, Risto Uusitalo, Jouni Lehtoranta, Helena Soinne, Jani Salminen

**Affiliations:** 1 Natural Resources Institute Finland, Luke, Latokartanonkaari 9, 00790 Helsinki, Finland; 2 Finnish Environment Institute (Syke), Latokartanonkaari 11, 00790 Helsinki, Finland

**Keywords:** Dissolved phosphorus, Efficiency, Environmental policy, Metric, Total phosphorus

## Abstract

Eutrophication of the Baltic Sea is driven by phosphorus and nitrogen. While the anthropogenic point source loads of both nutrients have decreased markedly, further reductions are needed. This is true particularly for phosphorus, as highlighted by its stringent abatement targets in HELCOM’s Baltic Sea Action Plan. To meet the targets, more results need to be achieved in non-point source abatement, specifically from agricultural sources. The growing pressure for phosphorus abatement from agriculture may lead to environmentally and economically inefficient outcomes unless we account for the variability in how different forms of phosphorus respond to abatement measures, and how these forms contribute to eutrophication. The precautionary and efficiency improving way to advance policies is to either replace or supplement the Total Phosphorus metric with a metric more accurate in reflecting the biologically available phosphorus. This policy fix becomes more important as the relative share of agricultural emissions of total pollution increases.

**Supplementary Information:**

The online version contains supplementary material available at 10.1007/s13280-023-01851-2.

## Introduction

The Baltic Sea used to serve as a recipient for untreated municipal and industrial wastewaters, until large-scale construction of treatment facilities during the second half of the 1900’s. Latest by then, the sea’s limited capacity to assimilate nutrient load had become apparent (Cederqvist et al. 2020). Protection efforts focusing on loading from point sources have successfully curbed N and P loading to the sea (Savchuk 2018). In agriculture, the extremely high N and P balances have been somewhat lowered since the turn of the millennium (Drohan et al. 2019; Eurostat 2022). Nevertheless, nutrient loading from non-point sources, particularly agriculture, continue to be high (HELCOM 2018). Therefore, eutrophication mediated by phosphorus (P) and nitrogen (N) continues to deteriorate the water quality of the Baltic Sea (Conley et al. 2009). Phosphorus loading is particularly excessive. This is highlighted by the Baltic Sea Action Plan (BSAP), the fulfillment of which still requires 31% abatement of P loading while N targets are met with a 6% reduction (HELCOM 2018). In addition to meeting the targets, we need to adapt to more accurate understanding of P as an agricultural pollutant and as a eutrophying substance. This is needed to improve the environmental and economic effectiveness of our P abatement efforts. Specifically, the current metric with which the abatement targets are set, *total P*, is a low-resolution alternative. It obscures important trade-offs in mitigating loading of phosphorus fractions with varying eutrophying impact. It, thus, hides pivotal complexities and makes agricultural phosphorus abatement seem more efficient than it is from the viewpoint of eutrophication.

As a body authorized by an international treaty, the Helsinki Commission (HELCOM) has a special role in the governance of the Baltic Sea. HELCOM also intermediates the science-policy interface (Tynkkynen et al. 2014) and is committed to use up-to-date scientific evidence. For example, the current nutrient abatement targets agreed by the parties are set in the Baltic Sea Action Plan (BSAP) which commits to *‘incorporate the latest scientific knowledge and innovative management approaches into strategic policy implementation’* (HELCOM 2021).

High abatement costs (Andersson et al. 2022), limited institutional and legal capacities of HELCOM (Kern 2011; Tynkkynen et al. 2014; Bohman 2018; Brady et al. 2022), and governance arrangements prevailing in the nine littoral states (Andersen et al., forthcoming) are identified as reasons for the gaps in the implementation of the HELCOM agri-environmental policies (Thorsøe et al. 2022). These studies discuss the institutional and political landscape of the Baltic Sea protection. However, they take the metrics with which the policy targets are set for granted. Yet these institutionalized policy parameters influence innovation, assessment, and ranking of cost-effective policy measures.

In this perspective article directed to scientists and policymakers involved in water and marine protection, we argue that together with the growing relative importance of non-point P loading, the policies promoting its abatement need to be re-evaluated. We start by outlining scientific fundaments regarding P and its different fractions and their role in eutrophication. Agricultural loading of the P fractions, their mitigation measures, and related trade-offs are reviewed as well. We then merge the above building blocks into a simplified efficiency analysis, emphasizing the importance of bringing understanding of the different P fractions concretely into the metric with which the abatement targets are expressed. Thereafter, we analyze the gradual separation of institutionalized P metrics and scientific understanding, and the policy implications of total P as the metric of HELCOM nutrient abatement policies. Finally, we suggest that the scientists studying P-driven eutrophication could start a process to agree on a new or additional metric that incorporates P fractions according to their eutrophying potential. In terms of concrete policy design, we also urge the policy institutions to adopt the present natural scientific understanding of the different P fractions, to set separate abatement and monitoring targets for them and renew the agricultural abatement strategies accordingly.

## Phosphorus as a driver of eutrophication

Phosphorus is a key nutrient in controlling the eutrophication of the Baltic Sea. The total P loading has decreased from its peak of about 70 000 tons a^−1^ in the late 1980s to less than 30 000 tons a^−1^ in 2017 (Savchuk 2018). Since the 1980s, the total phosphorus loading from coastal point sources has decreased from about 22 000 tons a^−1^ to 3000 tons a^−1^ (Savchuk et al. 2012). Consequently, the role of non-point source pollution has become more important, with the estimated share of anthropogenic non-point sources to the Baltic Sea at 60% of *riverine* loads (Sonesten et al. 2018). As the relative share of diffuse loading has increased, it has also meant that the particle-associated P fraction has become more dominant. At the same time, our understanding of the impacts of the different fractions of P has increased.

In the following chapters, we review this scientific knowledge and reflect it against the present abatement strategies targeting the eutrophication of the Baltic Sea. For clarity, we focus on the following main fractions of P: Total P (TP), Particulate P (PP), Dissolved reactive P (DRP), and Bioavailable P (BAP); we approximate that BAP is the sum of DRP and the bioavailable part of PP, and that TP = PP + DRP.^1^

### Phosphorus fractions and their fate in the Baltic Sea

Rivers carry phosphorus to the Baltic Sea in particulate (PP) and dissolved forms (DRP). These forms differ in their origin, fate, bioavailability, and abatement possibilities. DRP is mostly bioavailable and, thus, strongly promotes eutrophication. Iron and organic-bound P are the major labile forms of PP, while P bound to calcium (e.g., as apatite P of land-derived particles) is largely inert in sediments (Mort et al. 2009; van Helmond et al. 2020). PP loading, which can be mitigated relatively effectively with erosion control measures, thus, contributes only partly to BAP. Furthermore, erosion control measures tend to unintentionally increase DRP loading. Focusing only on erosion control may, thus, be ineffective, and in extreme cases, it may even promote eutrophication (Jarvie et al. 2017). The outcome depends on the strength of the erosion control—DRP loading trade-off and on the bioavailability of PP loading.

Upon entering the coastal waters, some of DRP is utilized by biota. Some of it may bypass the filtering zones and be transported to the open Baltic Sea outside the productive season. The open waters of the Baltic have very low iron concentrations (Gelting et al. 2010), and only a minor amount of DRP can be sequestered by iron in oxic surface waters, meaning that DRP migrating there remains largely available to biota. In contrast, PP is largely trapped in estuaries and coastal areas (Lukkari et al. 2008; Asmala et al. 2017; van Helmond et al. 2020). The ability of the bottom sediment to hold the settled particulate iron- and organic-bound P depends on four factors: (1) delivery of oxygen, (2) amount of labile organic matter, (3) amount of metal (Fe, Al, Mn) oxides in sediments, and (4) sedimentation rate (Mort et al. 2009; Lehtoranta et al. 2015; Asmala et al. 2017; van Helmond et al. 2020). Commonly the coastal surface sediments of the Baltic Sea retain P well due to their better oxygen conditions and higher iron oxide content than the sediments in the deep basins (Mort et al. 2009). In addition, settled particulate P also includes detrital P, calcium-bound P and other mineral structure-embedded P which is inherently recalcitrant, i.e., is dissolved very slowly, if at all, in the coastal and open Baltic Sea sediments (Mort et al. 2009; van Helmond et al. 2020). Therefore, expressed in terms of abatement, reducing DRP mitigates eutrophication with certainty, while reducing PP is less pivotal, especially in coastal areas where good oxygen conditions prevail in bottom waters.

In the 29 Finnish rivers discharging into the Baltic Sea, an average of 65% of the P transported is in the form of PP while the shares of DRP and dissolved unreactive P are both 18% (Räike, unpublished, data available in Finnish Environment Institute 2022). However, in some river basins, such as River Warnow in Germany, the share of DRP may reach 40% of total P (Rönspieß et al. 2020). Often comprehensive knowledge about the shares of the different P fractions is lacking because P forms are seldom included in the national monitoring programs (Jarosiewicz et al. 2015; HELCOM PLC data). In addition, when included the methodology is superficially described, for example, with regard to filtration that crucially affects speciation between particulate and dissolved fractions (Jarosiewicz et al. 2015; HELCOM PLC data).

### Agricultural P loading, mitigation measures, and their impacts on P fractions

For any given field parcel, agricultural P loading is roughly driven by the long-term soil P balance and the applied cultivation techniques, including agricultural water protection measures. Until the 1990s, soil P balances in developed countries typically showed large surpluses, up to tens of kilograms per hectare, due to the excessive use of mineral fertilizers (OECD 2022). Since then, mineral fertilizer application rates have diminished substantially. By 2018, in almost all countries around the Baltic Sea, the mean P surpluses have declined to about 5 kg ha^−1^ or less (OECD 2022). However, in areas with intensive animal husbandry, local overapplication of P persists due to the formation and spreading of large volumes of manure.

Soil holds finite amounts of P-binding constituents. If the applied P quantity is larger than plant P uptake, i.e., P is applied at surplus rates, P saturation on binding surfaces increases. Gradual increase in P saturation builds up a pool of readily soluble P in soil (Cope 1981; Cox 1992), a pool that is easily accessible for plants but also at risk of being lost to water bodies (Heckrath et al. 1995). Agronomic soil P tests that are used to indicate the amount of plant-available P in soils, capture the long-term changes in soil P pool that is labile enough to be dissolved in rain- and snow-melt waters. Soil P tests are, hence, used as an indicator for the potential for P losses, of DRP in particular (Schoumans and Groenendijk 2000; Dodd et al. 2012). Regulation of P fertilization based on field-parcel-specific soil P test values consequently also affects DRP loading. As for PP loading, erosion control is the most effective way of mitigation. It reduces the loading of eutrophying P, too, but only for the bioavailable fraction of PP.

Many widely promoted agricultural water protection measures targeting eutrophication typically encompass trade-offs between DRP and PP loads (Uusi-Kämppä et al. 2000; Tanner and Sukias 2011; Jarvie et al. 2017). A relevant example is long-term no-till practice, meaning cultivation of crops with minimal soil disturbance. On the one hand, no-till significantly reduces erosion and related loss of PP from fields outside the growing season. On the other, the practice results in a gradual P saturation of the uppermost surface layer of the soil (Thompson and Whitney 2000). This is driven by the combination of plant P translocation and possible surface P applications that are not mixed within soil. Plant P translocation occurs in all non-inverted soils regardless of P fertilizer application, also in soils not used for crop production. Plant roots extract soil P most vigorously at the depth of about 5–30 cm. Part of this P is translocated to the aboveground biomass and upon the decay of the plant residues P is released onto the soil surface. Freezing and thawing enhance the release of DRP from the plant residues (Bechmann et al. 2005; Liu et al. 2019). Hereby, the uppermost soil surface, typically not thicker than a few centimeters, becomes enriched in readily soluble P (Sharpley et al. 1978; Lozier et al. 2017). Substantial P stratification develops relatively quickly, within 3 to 5 years after plowing (Ellis and Howse 1980; Rhoton 2000; Li et al. 2019). Since the enriched surface soil is in contact with rain and meltwater, prolonged no-till practices and use of cover or catch crops without inversion tillage are likely to result in considerable increase in DRP loads even though PP loads remain stable (Sharpley and Smith 1994; Puustinen et al. 2005; Uusitalo et al. 2018).

### Lake Erie as an example of realized risks

Lake Erie offers an example and a warning sign for the Baltic Sea regarding unintended outcomes of large-scale land-management changes to mitigate TP loading. The example shows that the trade-offs in P mitigation are not merely academic oddities but must be taken seriously in policy design.

Lake Erie is one of the Great Lakes located between the US and Canada. In late 1960s, the TP load to the lake peaked at around 27 000 tons a^−1^ followed by a rapid decrease. In mid-1980s, the TP load averaged some 10 000 tons a^−1^. The achievement was attributed to reductions in point sources (Maccoux et al. 2016). Since the 1990s, the average TP loading has been relatively stable.

Despite TP loading remaining on a lower level, the status of the lake started turning worse after the mid-1990s, eventually leading to record-setting blooms of toxin-forming straits of blue-green algae, increased oxygen deficit in near-bottom water layer, and sediment P release (Michalak et al. 2013). As a potential explanation, P loads carried by individual rivers exhibited a disturbing pattern. Daloğlu et al. (2012) and Baker et al. (2014) reported dramatic increases in DRP loadings by the Maumee and Sandusky rivers since the mid-1990s. The increasing trend was linked to substantial changes in cultivation techniques in the watershed (Daloğlu et al. 2012; Michalak et al. 2013; Jarvie et al. 2017). Erosion control techniques such as no till had been favored to mitigate erosion and, thus, PP loading. As in the northern Baltic Sea, PP made up most of the TP load, and therefore, the efforts seemed justified. In the Sandusky, Maumee, and Raisin watersheds, soil surface preserving cultivation techniques covered 10% of agricultural land in 1980s. In 2012, their share had increased to 50–70%. DRP loading in these rivers increased in lockstep with this development, despite decreasing P balances (Jarvie et al. 2017). It should be noted that while the trade-offs of DRP and PP are similar in the source areas, the effects in the environment could be different in Lake Erie and in the Baltic Sea. The water residence time of Lake Erie, for instance, is less than three years while that of the Baltic Sea is about 30 years. However, as noted above, the sedimentation and burial of PP take places already in coastal areas and estuaries. In this sense, the aging of sediment is less different than the water residence time would suggest.

## Efficiency implications of the metric

The well-known trade-offs between the abatement of PP and DRP loading from agriculture, and the fact that a unit of DRP loading is substantially more harmful than that of PP, calls for a re-evaluation of the benefits of mitigating P losses by controlling erosion with permanently vegetated soil as a categorical nutrient abatement measure. The benefits of erosion mitigation are obvious and undisputable if soils without vegetative cover are heavily erodible. However, promoting permanent vegetation on fields with low inherent erosion risk is problematic as shown by the scientific evidence from Lake Erie catchment.

Thus far, the *metric* for the eutrophication abatement strategies in the Baltic Sea region has been TP. In the following, we exemplify the relevance of a more refined metric for the abatement targets. To illustrate this, consider a set of one hundred, one hectare-sized parcels with identical soil test P but with erodibility (PP loading) that increases in equal steps due to stepwise increasing slopes of the fields. Assume they are initially all plowed every year but can be converted to no-till to reduce erosion and PP loading. For illustration, we abstract away from any other impacts the choices may have and focus only on the losses of P fractions with runoff.

Utilizing the data of Uusitalo et al. (2018) and Puustinen et al. (2005), we first link no-till and plowing choices to parcel-specific PP and DRP losses (see Supplementary material for more details on the method). If plowed annually, the annual DRP loading from each parcel is 0.14 kg ha^−1^. Erosion susceptibility and thus PP loading rates differ between parcels, being distributed evenly between the extremes of annual PP loss of 0.5 to 5 kg ha^−1^. If plowed annually, the PP and DRP loadings from the region are 275 kg a^−1^ and 14 kg a^−1^, respectively, yielding a TP loading of 289 kg a^−1^. Assuming 25% of PP turns eventually into bioavailable form in the receiving waters, the BAP loading is 83 kg a^−1^.

**Fig. 1:**
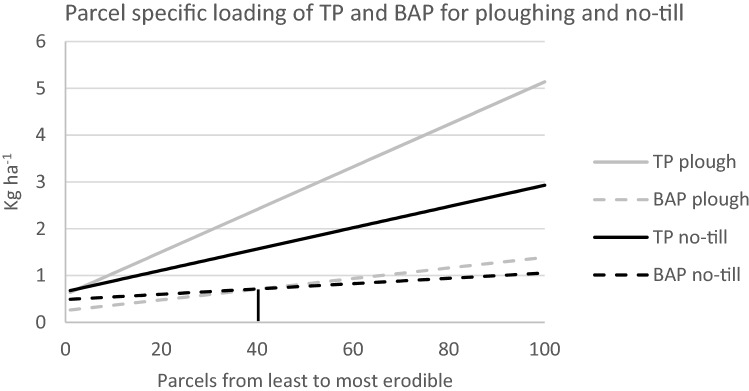
Total P (TP) and Bioavailable P (BAP) loading associated with ploughing and no-till for the 100 hectare sized parcels of our exemplifying watershed. Parcels ordered from the least to most erodible. The bioavailability of PP 25%

The parcels in Fig. 1 are ordered from the least to the most erodible. The gray solid line indicates the TP loading under plowing from each parcel, and the dotted gray line, the BAP loading (calculated for 25% PP bioavailability). The solid black line denotes TP loading under no-till and the dotted line BAP loading. The short vertical line indicates the threshold. To minimize BAP loading, parcels less erodible than this should be plowed, more erodible put on no-till.

Figure 1 depicts the parcels-specific TP and BAP loadings for plowing and no-till. Table 1 shows the optimal choices between these two, when two alternative targets are being pursued: minimization of TP or BAP loading.

**Table 1:** Average losses of particulate P (PP) and dissolved reactive P (DRP), and relative reductions of Biologically Available P (BAP) and Total P (TP) loading resulting from two alternative management strategies. Reductions calculated as a difference from the business-as-usual case where all hundred parcels are plowed

Management strategy	Plowed / no-till parcels	PP (kg a^−1^)	DRP (kg a^−1^)	TP (kg a^−1^)	BAP (kg a^−1^)	BAP reduction (%)	TP reduction (%)
*Business-as-usual*	100/0	275	14	289	83	–	–
*Minimize TP*	2/98	138	42	180	77	7	38
*Minimize eutrophying P*	41/59	166	31	197	73	12	32

Converting from annual plowing to no-till increases the annual DRP loading in 5–10 years to 0.43 kg ha^−1^ and cuts the PP loading to half (Uusitalo et al. 2018). Switching the entire region to no-till would, thus, decrease the TP loading to 181 kg a^−1^ and the BAP loading to 77 kg a^−1^. A target of minimizing the TP loading would leave the two least erosion susceptible parcels on plowing while converting the other 98 to no-till. This would result in a TP loading of 180 kg a^−1^ and a BAP loading of 77 kg a^−1^.

However, if we were to minimize BAP, we would have 59 parcels converted to no-till and 41 parcels on plowing. The TP loading would be 197 kg a^−1^ and the BAP load 73 kg a^−1^. The target of minimizing the TP loading would, thus, reduce BAP only about a half of what the strategy of minimizing eutrophying P loading would do. Two things are worth noting: first, the maximal reduction in TP loading is 38% while the maximal reduction of BAP loading is only 12%. The outcome, thus, looks more optimistic when looking at TP only. Second, the outcome is extremely sensitive to the assumption of bioavailability of PP. Table 2 summarizes the obtained reductions for three different PP bioavailability values when following either the current metric (maximal TP reduction) or the more precise eutrophying P metric.

**Table 2:** Obtained reductions when targeting either TP or eutrophying P reductions for PP bioavailability values 10%, 25%, and 50%. Negative values indicate increased loading of a P fraction

Metric of the target	Minimize TP			Minimize eutrophying P		
Reduction in terms of	TP (%)	BAP (%)	Area on no-till (%)	TP (%)	BAP (%)	Area on no-till (%)
10% Bioavailability of PP	38	− 35	98	0	0	0
25% Bioavailability of PP	38	7	98	32	12	59
50% Bioavailability of PP	38	27	98	37	27	85

If 10% of PP eventually turned bioavailable, the target of minimizing TP would *increase* the BAP loading by 35% while the target of minimizing eutrophying P would maintain the initial allocation and *not be able to reduce* BAP *loading at all.* If the bioavailability of PP is 50%, there are basically no differences in measure allocations. Table 2 shows two important things. First, optimal allocation of measures is strongly dependent on the bioavailability of PP. Second, basing the policy targets on the metric that reflects more accurately the environmental impact of the pollutant significantly reduces the uncertainty. If PP turns out to be more potent in terms of eutrophication than currently thought, the policies will nevertheless be correctly chosen.

In our example, soil erodibility increased linearly from one hectare to another, with PP losses increasing from 0.5 to 5 kg ha^−1^. If the landscape had no erodible parcels, TP minimizing policy would appear merely detrimental in eutrophication abatement. Then again, for a uniformly heavily erosive area, the two abatement strategies would result in almost similar choices of erosion control measures (see supplementary material for calculations).

Taking into account, N abatement would change the efficiency consideration. No-till and other vegetative cover practices reduce N loading and would therefore become more effective, if the receiving waters are sensitive to N loading. However, even when analyzing the joint effect of N and P, the eutrophying effect of P should be modeled as accurately as possible. This might mean that potential achievements in reducing eutrophying loading from agriculture would become even more difficult. This would shift the emphasis of efficient eutrophication management to, for instance, point sources which are typically considered too expensive for further reductions.

## The phosphorus understanding advocated by HELCOM nutrient abatement policies

How are the PP-DRP trade-offs and the fact that DRP is more potent a driver of eutrophication reflected in Baltic Sea protection policies? To capture the P understanding adapted by HELCOM, we analyze how the key HELCOM policy documents (i) parametrize P loading and (ii) take explicit or implicit stand on the priority of different P fractions.

Studies on the governance of nutrient abatement in the Baltic Sea region (Tynkkynen 2015; Bohman 2018; Thorsøe et al. 2022) emphasize two policy documents. The first is the Convention on the Protection of the Marine Environment of the Baltic Sea Area (Convention 1974). The second is the recently updated Baltic Sea Action Plan (BSAP).

### Convention

The Convention is a binding international agreement that leaves flexibility for the State implementation (Bohman 2018). The regulatory approach of HELCOM builds upon the Convention, including its amendment and Annexes. Agricultural provisions were first included in the updated Convention in 1998 and amended in 2007. Table 3 lists the agricultural measures of Annex III that the signatories have agreed to implement through their national regulations (Bohman 2018; Thorsøe et al. 2022).

**Table 3:** Convention provisions addressing agriculture

Number	Provision (for full descriptions see https://helcom.fi/about-us/convention/annexes-to-the-convention-2/annex-iii/)
1	Animal density: to ensure that manure is not produced in excess in comparison to the amount of arable land, there must be a balance between the number of animals on the farm and the amount of land available for spreading manure, expressed as animal density
2	Location and design of farm animal houses: farm animal houses and similar enclosures for animals should be located and designed in such a way that ground and surface water will not be polluted
3	Construction of manure storage. Manure storage must be of such a quality that prevents losses. The storage capacity shall be sufficiently large to ensure that manure only will be spread when the plants can utilize nutrients. The minimum level to be required should be 6 months’ storage capacity
4	Agricultural wastewater and silage effluents: wastewater from animal housing should either be stored in urine or slurry stores or else be treated in some suitable manner to prevent pollution
5	Application of organic manures: organic manures (slurry, solid manure, urine, sewage sludge, composts, etc.) should be used in such a way that a high utilization efficiency can be achieved
6	Application rates for nutrients: the application of nutrients in agricultural land shall be limited, based on a balance between the foreseeable nutrient requirements of the crops and the nutrient supply to the crops from the soil and the nutrients with a view to minimize eutrophication
7	Winter crop cover: in relevant regions, the cultivated area should be sufficiently covered by crops in winter and autumn to effectively reduce the loss of plant nutrients
8	Water protection measures and nutrient reduction areas: buffer zones, riparian zones or sedimentation ponds, permanent grassland, wetlands; establishing groundwater protection zones, and nutrient reduction areas
9	Ammonia emissions: for example, surplus of nitrogen in manure should be avoided by adjusting the diets of individual animals; urine and slurry storages should be covered

Many of the measures are in line with the codes of good agricultural practice as defined by the EU Nitrates Directive. However, the Convention applies to entire national territories in the littoral states, including Russia, and to regions within member states that are not designated as nitrate vulnerable zones. Some of the HELCOM measures are also stricter than the codes defined by Nitrates Directive. (Thorsøe et al. 2022.)

The Convention also imposes explicitly P-oriented measures. *Proportioning of animal density to the amount of arable land* seeks to balance manure nutrient applications with crop needs. The requirement of *nutrient application rates* then reflects a P understanding that acknowledges the importance of the increased risk for DRP losses caused by overfertilization and increasing soil P stocks.

The Convention requires the use of *Winter crop cover* ‘to effectively reduce the loss of plant nutrients’ (Convention p. 25). Nutrient losses are to be prevented also with the means of *buffer zones, riparian zones, sedimentation ponds* and *wetlands*. The obligations are to be applied in unspecified ‘relevant conditions.’ The advocating of winter vegetation cover is likely emphasized as a N loss mitigation method, but regarding P losses it either prioritizes erosion prevention from the outset or, alternatively, dismisses the findings indicating increased DRP losses from fields maintained vegetated during winter. Likewise, permanent grasslands and buffer zones presume reduced soil disturbance that will lead to enrichment of uppermost soil surface layer with P as described in Section “Phosphorus as a driver of eutrophication”.

According to Thorsøe et al. (2022), winter vegetation cover and water protection measures such as buffer zones and wetlands are relatively well instituted to the policy frameworks adapted in HELCOM countries. In Finland, Sweden, and Poland, the measures combatting erosion and PP loading have been defined as key means of eutrophication prevention in the River Basin Management Plans (Piniewski et al. 2021). The emphasis given to erosion prevention measures in the Plans is problematic also from the inland waters point of view. In rivers and lakes, phosphorus tends to be the limiting nutrient. This implies that acknowledging the trade-offs between DRP and PP would be beneficial for inland waters as well.

The Convention is relatively old and cannot be expected to reflect the latest scientific findings. However, it is worth noting that scientific discussion about the role of bioavailability of PP and the impact of cultivation practices on different P fractions abounded already well before 2007 when the Convention Annex III was amended (see, e.g., Johnson et al. 1979).

### BSAP

The BSAP was first adopted in 2007 with the most recent update launched in Autumn 2021. It constitutes ‘HELCOM’s strategic programme of measures and actions for achieving good environmental status of the sea’ (HELCOM 2021). Although the legal status of the BSAP is unclear (Bohman 2018), it sets the environmental targets to be attained through contracting parties’ national policies.

The goal of the plan is good environmental status of marine waters. Taking into account the current status of eutrophication, nutrient loading pressures, and the sensitivity to eutrophication, the BSAP defines basin-specific maximum allowable nutrient inputs and their reduction targets (Bohman 2018; HELCOM 2021). These are further allocated as net nutrient input ceilings (NIC) for each country, expressed using the metric total N and total P (tons a^−1^).

The BSAP follows the open-ended regulatory approach familiar from the Marine Strategy Directive and the Water Framework Directive (e.g., Valve et al. 2017; Bohman 2018), leaving the means of nutrient abatement to littoral states. The most recent BSAP reinforces the prevailing P understanding, although it introduces some new measures. One of these is gypsum amendment, a measure potentially mitigating both PP and DRP loading (Ekholm et al. 2012; Uusitalo et al. 2012). The BSAP does not, however, take stance whether the action to be taken focus on prevention of PP or DRP. The target metric *implicitly encourages measures focusing on PP abatement as its share of TP is higher*.

## Discussion and recommendations

The evidence of the scientific literature is compelling: DRP has a considerably higher eutrophying potential than PP, and there are trade-offs in DRP and PP abatement from agricultural non-point sources. In the Baltic Sea, this scientific understanding and the protection policies have, however, diverged. In the core of this decoupling is the metric, the most fundamental policy targets by HELCOM are bound to. HELCOM abatement strategies and monitoring rely on TP and, thus, disregard the trade-offs between PP and DRP, creating a risk for increased DRP loads.

The above-mentioned decoupling entails that governance research focusing on nutrient abatement policies and protection of the Baltic Sea must adopt a critical perspective towards the metrics on which the governing system (Kern 2011; Tynkkynen et al. 2014) operates. Taking institutionalized policy parameters for granted is ill equipped to support adaptive governance of the Baltic Sea (see Heiskanen et al. 2019; Grönholm 2020). In an extreme case, social science research may even contribute to path-dependence and act against reassessment and reflection.

Although the ‘control panel’ through which policy steering occurs typically adapts to new scientific knowledge with delay, there are reasons for serious concerns if the discrepancy between policy and scientific understandings becomes institutionalized. This diverges the realms of science and policy, reducing potential for a reflexive dialogue. To facilitate the necessary change in the science-policy interface related to the metric applied in the nutrient reduction targets, both the policy institutions governing the Baltic Sea protection—most notably HELCOM and the EU Commission—and the scientific communities analyzing the ecosystems of the Baltic Sea need to make contributions. Additionally, learning from policy innovations and metrics therein established in other intensively protected water bodies like Lake Erie merit attention.

A first concrete option to incorporate updated knowledge in water protection with a focus on eutrophication would be to start a process for defining the concept of P equivalents (Iho et al. 2017). This could be done in the same manner as CO_2_-equivalents which commensurate various greenhouse gases based on their defined climate impact (MacKenzie 2009). CO_2_-equivalents prevent unintended trade-offs potentially emerging, for example, when decreasing two tons of carbon dioxide emissions increases a ton of methane emissions. In this paper, we exemplified the introduction of such a metric into the management of fields to optimize the reduction of eutrophying P to the Baltic Sea. Currently there is a lack of means that can support cost-effective improvement of the eutrophication status of the Baltic Sea (Helcom Science Agenda 2021).

Scientific communities—including social scientists—should also be active in interceding the natural scientific and policy-related understanding from other heavily protected water bodies to the context of the Baltic Sea and its protection. In the Lake Erie protection policies, the dilemma with the trade-off has been acknowledged. The Lake Erie Management Program from 2015 suggests that only 25–50% of PP may be considered bioavailable P (BAP), which emphasizes the importance of mitigating DRP loading (USEPA 2015). The U.S. Action Plan for Lake Erie (USEPA 2018) sets explicit targets for DRP loading from the Maumee and Sandusky Rivers. Also, the Canada-Ontario Lake Erie action plan aims at 40% reduction from the 2008 levels for both TP and DRP by 2025 (Ontario Ministry of the Environment and Climate Change 2018). In the Chesapeake Bay, the Scientific and Technical Advisory Committee suggested that state specific targets should be set in terms of eutrophying units as soon as feasible (STAC 2019). In our view, the protection policies and related metrics applied in Lake Erie and Chesapeake Bay provide an example that could be adopted in the context of the Baltic Sea, too. This way, the obvious risk for failures in protection policies of the Baltic Sea similar to those experienced in Lake Erie could be reduced.

Second, based on the natural scientific evidence and the findings from Lake Erie, we propose the following. All HELCOM countries should be required by HELCOM to evaluate their implemented agri-environmental measures’ impacts on DRP and PP loadings and also strive towards including the P fractions in the nutrient load monitoring and reporting. This would not require much extra work as the EU member states have to submit their CAP strategy programs, including accounts of the agri-environmental measures to be subsidized, to the EU Commission anyway. The programs include estimates of the abatement effects of different measures on TP. Their effects on DRP and PP are relatively well known for the most important measures.

In the re-parametrization of nutrient abatement policies, HELCOM holds a key position. It performs significant coordinative work across HELCOM countries and, while being committed to the utilization of best available science, translates scientific findings into marine protection policies. Due to its science-based authority, HELCOM can be more influential than its limited legal powers would indicate. Although the HELCOM parties do not implement the regulations in full, it is unlikely they would adapt protection measures that are radically in conflict with the HELCOM policy framework. Furthermore, as an organization that actively feels the pulse of science and scientists, it can shape policy-making not only through the regulations to which the HELCOM countries are committed, but also signal needs for change beyond its jurisdiction.

## Concluding remarks

So far, the protection of Baltic Sea has been successful in terms of cutting down the loading of TP. However, as the relative importance of non-point loading increases, the Baltic Sea should be protected against similar setbacks as experienced with agricultural P loading to Lake Erie. To this end, scientists should agree on a new or additional metric that incorporates P fractions according to their eutrophying potential. The policy institutions—first and foremost HELCOM and the EU Commission—should adopt the improved aquatic, soil, and agricultural scientific understanding of P and eutrophication.

## Supplementary Information

Below is the link to the electronic supplementary material.

Supplementary file1 (PDF 1530 kb)

## Footnotes

1 This speciation of P is not exhaustive. For a more thorough and accurate one, see Supplementary material, Table S1.
