# Supplementary material for: Efficient protection of the Baltic Sea needs a revision of phosphorus metric
Source: Ambio. 2023 Apr 10;52(8):1389–99. doi: 10.1007/s13280-023-01851-2 (PMC10271980; doi:10.1007/s13280-023-01851-2)
Supplement: Supplementary file 1 — Supplementary file1 (PDF 1530 kb) [file 13280_2023_1851_MOESM1_ESM.pdf]

Ambio

Electronic Supplementary Material

This supplementary material has not been peer reviewed.

Title**: Efficient protection of the Baltic Sea needs a revision of phosphorus metric**

Authors: Antti Iho, Helena Valve, Petri Ekholm, Risto Uusitalo, Jouni Lehtoranta, Helena Soinne, Jani Salminen

Phosphorus analyses

In general, P entering surface water bodies appears in particulate and dissolved forms (for summary, see Table S1). In routine monitoring, TP is determined by digesting a sample in an acidic or alkaline environment and analyzing the P that reacts with molybdenum to give the more intensive color the higher is the P concentration (Table S1). Although called *total* P, the determination may exclude some of the P present in mineral or organic matter (Turtola 1996).

From a practical viewpoint, using TP as a criterion is reasonable as it is the most analysed P species in e.g., riverine waters, i.e., there is plenty of data of the TP loading. The determination does not mimic any biological uptake mechanism. By contrast, algae can ubiquitously use in their growth only the P fraction called dissolved orthophosphate. With few exceptions, all other P forms must be transformed into dissolved orthophosphate before being able to pass through the cell wall (Cembella et al. 1984). Determining dissolved orthophosphate requires a special analytical procedure, not suitable for routine water analysis. Algal assays have shown that the P species passing a filter and reacting with molybdate (i.e., DRP) are essentially available to algae in agricultural runoff (Lee et al. 1980, Ekholm and Krogerus 2003). In turn, DUP, the fraction that goes through a filter but requires digestion to react with molybdate is sparingly available (Lee et al. 1980, Ekholm and Krogerus 2003). The matter retained by a filter (particulate P) is partly transformed into an available form in water and sediments, but the available fraction depends on the characteristics of soil and receiving water and is somewhat unclear (Lehtoranta et al. 2015).

Although analytically straightforward, TP is seldom differentiated into the above fractions in national river monitoring programs in the Baltic Sea countries. For example, Sweden analyses only P from unfiltered samples (reactive P, RP) in addition to total P. The interpretation of this P fraction is the more problematic the richer the sample is in inorganic particles. Although the determination of RP does not have a digestion phase, the color formation phase is facilitated by ascorbic acid and sulfuric acid that transform originally unreactive, redox- and acid-sensitive P forms into reactive ones.

Table S1. Description of different P fractions and the chemical analyses to measure them. The abbreviations used for the different fractions are indicated in parentheses.

**P fraction**	**Description and remarks on chemical analysis**

Total P (TP)	- Most P present in a water sample.<br>- Analysis consists of three steps: (1) digestion (heating, oxidizing and acidifying the sample) transforms majority of P species into orthophosphate that after (2) staining gives a colored substance of which intensity is (3) measured spectrophotometrically.
Dissolved total P (DTP)	- Orthophosphate, condensed P and colloidal inorganic or organic P that pass a filter (pore size commonly ~0.4 µm).<br>- Analysis includes filtration followed by the analysis of P in filtrate (as above).
Particulate P (PP)	- P on and in mineral particles and organic matter, and P in larger precipitates.<br>- PP is obtained as TP minus DTP.
Dissolved reactive P (DRP)	- Orthophosphate plus condensed P and colloidal P made reactive by an acidic and a reducing reagent. DRP in agricultural runoff is mostly bioavailable.<br>- Analysis includes filtration, staining and measurement.
Dissolved unreactive P (DUP)	- Condensed P and colloidal inorganic or organic P that pass a filter (pore size commonly 0.4 µm) but require the digestion step to react in the chemical analysis. For example, humic-bound P.<br>- DUP is obtained as DTP minus DRP.
Bioavailable particulate P (bPP)	- PP that turns into a bioavailable form in an aquatic system over time. The transformation depends on the chemical nature of the PP (e.g. adsorbed on particle surfaces vs. embedded in mineral structures) and is affected by many processes during the transport from the source to deep sediments.<br>- There is no widely adopted routine analyses for this fraction. Estimates based on biological (algae or bacterial tests), chemical extractions (e.g. with NaOH or buffered dithionite) and using P sinks (anion exchange resin, FeO impregnated paper) suggest that typical range for bPP in agricultural runoff is 20-60% of PP.

Bioavailable P (BAP)	- P already being or eventually becoming biologically available to aquatic organisms.<br>- BAP is the sum of DRP and bPP and can be regarded as a theoretical concept due to problems in determining bPP.

On the release of sediment PP

PP can be transformed into a bioavailable form during the transport from source, after entering the receiving waters and finally in bottom sediments. What happens after settling to PP and its binding agents dictates the amount of bioavailable P released. Most of the sediment PP that becomes bioavailable is bound to organic matter and iron (Fe) oxides. Organic P is mineralized and released as DRP in respiration through oxygen (O_2) and other terminal electron acceptors such as nitrate (NO_3), iron oxides, and sulfate (SO_4). Yet, the released DRP may be efficiently scavenged by Fe oxides and the availability of oxides is associated with low release of P. However, P bound to Fe oxides can be set free when Fe oxides are reduced in anaerobic microbial or chemical reduction. There is a distinct difference between these two reduction reactions. Microbial Fe reduction forms dissolved reduced Fe, which may migrate upwards and create new P binding Fe oxides in presence of O_2. The chemical reduction, in turn, occurs through SO_4 reduction that generates sulfides which build solid Fe sulfides unable to bind P. What follows is that Fe sulfides remain in sediment, but dissolved P is able to escape from sediment to water. Respiration of Fe oxides and SO_4 are both microbial processes and fed by organic C and these processes largely dictate the behavior of sediment PP.

There is a great variation in the release of DRP from PP between the sub-basins of the Baltic Sea. In the Bothnian Bay sediment can retain P well due to good O_2 conditions, low organic C content, and high amount of Fe oxides in sediment. In the main basin of the Baltic Sea strong stratification maintains deep water anoxia, which favors SO_4 reduction and formation on Fe sulfides. In the main basin most of the PP bound to organics and Fe oxides will be released as bioavailable P in seawater.

P loading fractions and cultivation methods

There are both indisputable and controversial links between P loading and agricultural practices. The clearest and most generally accepted link is that between high long-term P balances, buildup of soil P and

elevated loading of DRP. It is also generally understood that erosion control and mitigating PP loading are tightly linked.

The controversial link is between the permanent plant cover, soil P stratification and the increase in DRP loading. As we will see, it creates constraints for effective P mitigation from agricultural lands.

Phosphorus builds up in the soil and increases DRP losses

P in terrestrial soils is relatively immobile and its inputs to the soil tend to be rapidly captured by the soil minerals, more specifically by the oxides of aluminium (Al) and iron (Fe), or in calcareous systems by calcium present in the soil. When the inputs in fertilizers and manure, or in any other P source, exceed the outputs by harvest, soil P concentration gradually builds up. How much a soil can bind P depends on its inherent properties such as the amount of Al and Fe oxides, or the activity of Ca^{2+} ions in soil solution. Accumulated P also gradually transforms to more stable forms. Agronomic soil P tests, primary aim of which is to assess the need for annual P fertilization, are also linked to the P build-up. They are therefore valuable indicators of P accumulation and widely used in environmental risk assessment.

There are readily available data on country-scale P inputs as fertilizers and manures and outputs via harvested yields (OECD, 2022). Their difference tells the P balances to agricultural soils over time. Soil P balances in developed countries typically showed large surpluses until 1990's, after which a clear reduction has occurred. By 2018 in almost all countries around the Baltic Sea the mean P surpluses have declined to about 5 kg ha^{-1} or less (Fig. OECD). Even negative P balances belong to sustainable nutrient management during the gradual depletion of legacy soil P.

Even with the lower P inputs since 1990’s, cumulative P balances indicate that P accumulation has continued to the present day in all of the other Baltic Sea countries except Estonia (-57 kg ha^{-1} in 2004-2015; or -4.8 kg (ha yr)$^{-1}$). Recent P balances show highest accumulations in Denmark (315 kg ha^{-1}, 1990-2015), Finland (257 kg ha^{-1}, 1990-2017; 9.2 kg (ha/yr)$^{-1}$) and Poland (121 kg ha^{-1}, 1990-2018; 4.2 kg (ha yr)$^{-1}$). P output in harvested yields typically remove P at a rate of 10-20 kg (ha y)$^{-1}$.

Suitable policies to adjust P inputs depend on the form in which the fertilizers are applied. In Latvia and Lithuania, for instance, mineral fertilizers make about 60-70% share of applied P. In Denmark, most P is applied with animal manure (Klinglmair et al. 2015). Mineral fertilizers are costly inputs for farmers and given that they understand the crop responses, it is in their economic interest to use them prudently. Manure, on the other hand, is abundantly available in the intensive animal production areas and its hauling cost often exceed its nutrient value. The economics of manure use generate areas with significant

overapplication and legacy P problems hard to tackle with regulatory tools (Niskanen et al 2020; Valve et al., 2020).

Decreasing N fertilizer use is another trend that we note from fertilizer sales statistics. Nitrogen has a hugely greater effect on yields than P, so decreasing N use may affect P use efficiency, i.e. the ratio between P removed and P applied. Lowering N applications more than P applications is likely to decrease P removal by harvest if the (mostly N-determined) yield potential is not achieved. Sufficient N fertilization is therefore a requirement for good P use efficiency. There are even some experiments on phytoextraction, i.e., maximizing crop yields even with very high N fertilization to accelerate legacy P depletion (Dodd et al 2014; Van der Salm et al 2009).

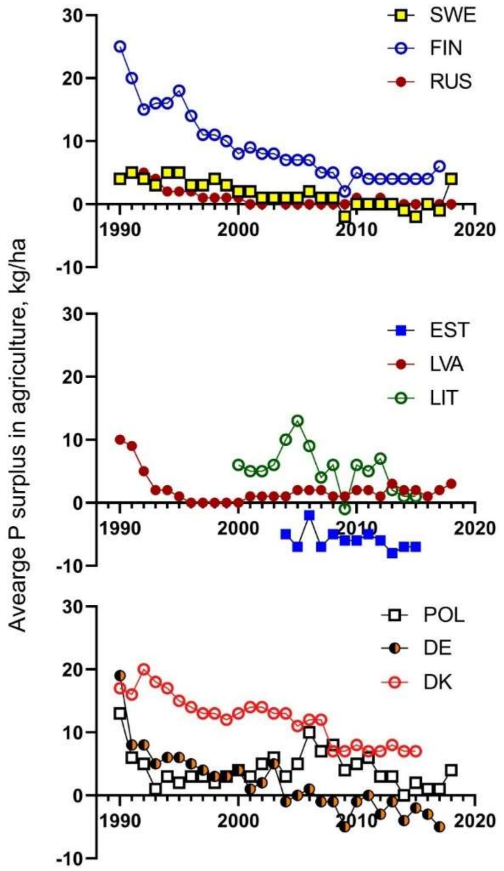

Fig. S1. Country-wise P balances around the Baltic Sea. Data obtained from OECD statistics (https://stats.oecd.org, Agri-environmental indicators).

There are little available data on the development of soil test P concentrations in the Baltic Sea countries. To our knowledge, the best coverage is from Finland with almost 1 million P test results for each of the Common Agriculture (CAP) program periods since 1996 (Lemola et al., 2018). As a result of decreasing P balances in Finland, soil test P at present (2015-2019) is on average 25% lower than in 1996-2000 period (Lemola et al., in prep.). Based on the data presented by Eriksson (2021), the development has been identical in Sweden. The Swedish country-wide sampling consists of about 2000 agricultural fields, the first sampling being conducted in 1988-1997. By the end of the third sampling round (2011-2017) mean soil test P (P-AL; ammonium lactate extractable P) was 24% lower than the mean of the first sampling. For Denmark, on the contrary, soils analysed in their occasional nation-wide grid sampling (about 340 sites) revealed a mean increase in topsoil (0-25 cm) Olsen P (sodium bicarbonate extractable P) by 6% and subsoil (25-50 cm) Olsen P by 20% between years 1987 and 1998 (Rubæk et al., 2013). Since the last soil sampling date, the Danish P balance has halved and the pace of increase has likely decreased – however, we note that the Danish P balance data still show almost 10 kg annual surplus.

DRP losses increase with increasing soil test P of a field parcel (Vadas et al. 2005). This phenomenon is universal, albeit not uniformly strong around the globe due to differences in soil test P analysis and soil properties (Withers et al., 2019). In catchment scale, soil test P may not emerge as a single explaining variable for P losses, because of several reasons, most of all due to stochasticity and uneven contribution of parcels to P loss events that overrides more subtle influence of many contributing factors (Kaushal et al. 2014). STP explain the DRP losses, but soil texture and management govern the mobilization of PP (Djodjic, et al 2018).

Erosion control and PP losses

For TP losses, erosion is the most important explaining factor and erosion-associated P (PP) often makes the main part of TP losses. Also here STP concentration affects PP losses, because P content of eroded particles gets higher with elevation of soil test P. The export of PP can be reduced with measures that increase soil aggregate stability and thereby prevents detachment of soil particles. The aggregate stability is better in soils with higher soil organic matter (Tisdall & Oades 1982, Soinne et al. 2016) and the risk for erosion is reduced as the soil clay/OC ratio decreases (Dexter et al. 2008, Schjønning et al. 2012, Soinne et al. 2016). However, regular disturbance of soil by ploughing weakens soil aggregate stability even in high OC soils (Soinne et al., 2016) highlighting the role of reduced tillage in erosion control. Furthermore, plant cover protects surface soil from raindrop impact that can detach soil particles from unprotected soil.

The different mobilization mechanisms of DRP and PP call for different measures to prevent their export. Minimizing soil tillage and the use of cover and catch crops are known to reduce soil erosion and are thus effective in reducing PP loading into surface waters. However, in fields with reduced tillage or permanent plant cover, higher loads of dissolved P have been reported.

P policies promoted by HELCOM

Table S2. A summary of measures affecting agricultural loading of DRP and PP.

Measure	Effect on PP, DRP
E5 Implement and enforce the provisions of part 2 of Annex III “Prevention of pollution from agriculture” of the 1992 Helsinki Convention.	
E6 Establish site specific buffer zones to reduce nutrient losses from agricultural land, for example on parts of fields where surface run-off and erosion occurs, along ditches or at surface water inlets.	Reduces PP, considered to increase DRP (Dosskey 2001, Uusi-Kämppä et al., 2000). Only a local impact.
E7 Balance fertilization rates site specifically and promote precision fertilization practices to improve nutrient use efficiency and reduce nutrient losses.	The most unambiguous measure in terms of decreasing the long-term risk for DRP loading. Affects the bioavailability of PP by gradually lowering the amount of soluble P in soil constituents
E8 Develop by 2025 and apply by 2027 the best practices to improve soil structure and aggregate stability on clay soils to reduce phosphorus losses from agricultural lands, for example by using soil structure lime or gypsum.	Affects both DRP and PP loading
E9 Promote organic farming to increase its proportion to at least 25% of agricultural land by 2030.	No direct effect on PP or DRP-loading. However, an indirect effect if associated with lowered spatial overapplication of manure in dense animal production areas. Organic farming increases the demand for organic fertilizers and this may be met with supply from animal production areas, reducing overapplication, gradually lowering soil P values and the risk for DRP loading. However, due to lower per ha yields from organic farming, the DRP and PP loading per produced agricultural products may increase.

E10 Discourage application of manure and other organic fertilizers in the autumn at fields without green plant cover in winter.	As such discouraging autumn spreading is beneficial. However, allowing it for fields with green cover is detrimental. Green cover does not help prevent losses from manure surface application outside growing season.
E11 Improve knowledge exchange by establishing dialogue between farmers, authorities and decision makers**.**	Effects of information guidance and education have been meager (e.g., Ribaudo & Horan 1999).
E12 Enhance mutual learning among farmers on best practices and innovative technologies.	As above
E13 Develop by 2025 recommendations for Best Available Technology (BAT)/Best Environmental Practice (BEP) to reduce ammonia and greenhouse gas emissions from livestock housing, manure storage and spreading.	Related to N emissions. Aillery et al (2005) show that mitigating air emissions from manure applications may increase P surpluses (by allowing higher application rates that comply with N balance requirements).
E14 Develop by 2025 recommendations for manure management specifically for horses, sheep, goats, and fur farming.	May affect direct losses and if an effect on application rates and decrease local overapplication, decrease long-term risk of DRP loading
Apply as a minimum the updated EU's Best Available Techniques (BAT) Reference Document and Conclusions on BAT for intensive rearing of poultry and pigs, especially for the facilities located within areas critical to nutrient losses.	Generally, technological regulation may be effective in preventing nutrient loading events from large animal facilities, particularly related to manure management and storage (e.g. Ribaudo et al 2003)
E16 Review national regulation and voluntary measures and – if relevant – implement further or revised measures, as compiled in the revised palette of measures for reducing phosphorus and nitrogen losses from agriculture.	A general action related to ensuring sufficiency of existing measures with no direct effects on either DRP or PP loading.
E17 Agree on national level by 2023 on measures to reduce nutrient surplus in fertilization practices to reduce nutrient losses.	Contributes to the long-run goal of balancing nutrient applications and uptake, and thereby gradually contributes to decreased loading of all P fractions. However, important to notice the joint effect of N and P. If implementing E17 causes N deficiency and thereby reduces yields and P uptake, may impede the long-term target (van der Salm et al. 2009)
E18 Investigate opportunities for taxation of mineral fertilizer and/or taxation of nitrogen surplus and/or payments for agri-environment	Here the risk of generating the unintended effects of diminishing crops yields due to N deficiency even more pronounced. In planning this measure one

measures by 2024 and implement them building on the experiences available in various countries.	should take into account the interdependence of N- and P-surpluses.
E19 Apply innovative water management measures where appropriate, for example, lime filter ditches, sediment traps and controlled drainage, and nature-based solutions, such as two-level ditches and constructed wetlands, when upgrading and renovating agricultural drainage systems.	Some measures influence both fractions, others (such as sediment traps) only PP-loading. We are not aware of studies analyzing the effects of compound channels on the dissolved nutrient fractions.

Efficiency Implications of a metric

Fig. S2 illustrates the simple optimization routine with which the efficient allocations between ploughing and no-till were calculated. The parcels have characteristic PP-loading values for ploughing, cut down by 50% if converted to no-till. Each parcel has an initial DRP loading value of 0.14 kg ha^{-1}, elevated to 0.43 kg ha^{-1} if converted to no-till. Here, TP is the sum of these two and BAP is the sum of DRP and the bioavailable fraction of PP.

Fig. S2. The binary choice of ploughing and no-till.

Parcel	Choice	DRP	PP	TP	BAP
1	Plough	0.14	0.50	0.64	0.24
	No-till	0.43	0.25	0.68	0.48
2	Plough	0.14	0.55	0.69	0.25
	No-till	0.43	0.27	0.70	0.485
. . .					
100	Plough	0.14	5	5.14	1.14
	No-till	0.43	2.5	2.93	0.93

The optimization procedure runs as follows. First, we select the PP-bioavailability (a value 20% used to generate the figure above). Then, we select whether we wish to minimize the sum of TP or the sum of BAP from all the parcels. If we minimize TP, the optimization routine chooses ploughing or no-till for each

of the 100 parcels to minimize the sum. Alternatively, we can set the sum of BAP as the target of minimization process.

From ploughing to no-till on uniform landscapes

In the base case, we used an evenly distributed erosion susceptibility between the lowest and highest PP loading values of 0.5 and 5kg ha^{-1}. In any given agricultural region, the distributions will be different. As extreme cases, consider two regions having oppositely erodible fields. Suppose that the uniformly flat region had the lowest PP loading values of our example for all fields 0.5 kg ha^{-1}, and the uniformly steep area the highest, 5 kg ha^{-1}. In both regions the initial cultivation method is ploughing. Table S3 collects the results for PP bioavailability values of 10%, 25% and 50%.

Table S3. From no-till to ploughing on two landscapes: uniformly flat and uniformly steep. Obtained reductions in terms of TP and BAP for PP bioavailability values 10%, 25% and 50%. Two landscapes: all flat or all steep. Negative values indicate increase in P losses.

Reduction from ploughing, in terms of	All on no-till, flat landscape		All on no-till, steep landscape	
	TP	BAP	TP	BAP
10% bioavailability of PP	-6%	-140%	43%	-6%
25% bioavailability of PP	-6%	-86%	43%	24%
50% bioavailability of PP	-6%	-42%	43%	36%

If the initial erosion is very low to begin with, moving to no-till increases the loading regardless of the metric used, i.e. even for TP. This is because the initial DRP loading is increased more than the initial PP loading reduced (DRP increased from 0.14 kg ha^{-1} to 0.43 kg ha^{-1} and PP reduced from 0.5 kg ha^{-1} to 0.25 kg ha^{-1}). The detrimental effect of the switch to no-till in terms of BAP loading is the stronger, the lower the bioavailability of PP. If only 10% of PP is bioavailable, BAP loading is increased by whopping 140%.

On the other hand, for uniformly steep landscape, converting the entire landscape to no-till is always the right policy if measured in TP loading (a decrease of 43%). It is also eutrophication mitigating option for PP bioavailability values of 25% and 50%. If only 10% of PP becomes bioavailable, no-till is associated with higher BAP loading than ploughing. In our example, the cutoff value is 12%. If the bioavailability of PP is

above this, no-till is unambiguously better in reducing eutrophication than ploughing for the uniformly steep region.

References

Aillery, M. P., N. R. Gollehon, R. C. Johansson, J. D. Kaplan, N. D. Key, and M. Ribaudo, 2005. Managing manure to improve air and water quality (No. 1477-2016-121076).

Cembella AD., N. J. Antia, and P. J. Harrison. 1984. The utilization of inorganic and organic phosphorous compounds as nutrients by eukaryotic microalgae: a multidisciplinary perspective: part 1. *CRC Critical Reviews in Microbiology* 10(4): 317–391.

Dexter, A. R., G. Richard, D. Arrouays, E. A. Czyż, C. Jolivet, and O. Duval. 2008. Complexed organic matter controls soil physical properties. *Geoderm*a 144(3–4): 620–627.

Djodjic, F., H. Elmquist, and D. Collentine. 2018. Targeting critical source areas for phosphorus losses: Evaluation with soil testing, farmers' assessment and modelling. *Ambio* 47(1): 45–56.

Dodd, R.J., R. W. McDowell, and L. M. Condron. 2014. Manipulation of fertiliser regimes in phosphorus enriched soils can reduce phosphorus loss to leachate through an increase in pasture and microbial biomass production. *Agriculture, ecosystems & environmen*t 185: 65–76.

Dosskey, M. G. 2001. Toward quantifying water pollution abatement in response to installing buffers on crop land. *Environmental Management* 28(5): 577–598.

Ekholm, P. and K. Krogerus. 2003. Determining algal□available phosphorus of differing origin: Routine phosphorus analyses vs. algal assays. *Hydrobiologia* 492: 29–42.

Eriksson, J. 2021. Tillståndet i svensk åkermark och gröda. Data från 2011-2017. Uppsala: Sveriges lantbruksuniversitet. *Ekohydrologi* 168.

Kaushal, S. S., P. M. Mayer, P. G. Vidon, R. M. Smith, M. J. Pennino, T. A. Newcomer, S. Duan, C. Welty, and K. T. Belt. 2014. Land use and climate variability amplify carbon, nutrient, and contaminant pulses: a review with management implications. *JAWRA Journal of the American Water Resources Association* 50(3): 585–614.

Klinglmair, M., C. Lemming, L. S. Jensen, H. Rechberger, T. F. Astrup, and C. Scheutz. 2015. Phosphorus in Denmark: national and regional anthropogenic flows. *Resources, Conservation and Recycling* 105: 311–324.

Lee O. F., R. A. Jones, and W. Rast. 1980. Availability of phosphorus to phytoplankton and its implications for phosphorus management strategies. In *Phosphorus management strategies for lakes*, ed. R. C. Loehr, C. S. Martin, and W. Rast, 259–308. Ann Arbor Science, Ann Arbor.

Lehtoranta, J., P. Ekholm, S. Wahlström, P. Tallberg, and R. Uusitalo. 2015. Labile organic carbon regulates the phosphorus release from eroded soil transported into anaerobic coastal systems. *Ambio* 44: 263–S273.

Lemola, R., R. Uusitalo, J. Hyväluoma, M. Sarvi, and E. Turtola. 2018. Suomen peltojen maalajit, multavuus ja fosforipitoisuus: Vuodet 1996–2000 ja 2005–2009. (In Finnish.) *Luonnonvara ja biotalouden tutkimus*. Natural Resources Institute Finland, Helsinki. http://jukuri.luke.fi/handle/10024/541851

Niskanen, O., A. Iho, and L. Kalliovirta. 2020. Scenario for structural development of livestock production in the Baltic littoral countries. *Agricultural Systems* 179: 102771.

OECD, 2022. OECD.Stat. Agri-Environmental indicators: Nutrients: Phosphorus balance. https://stats.oecd.org/index.aspx?queryid=79765 (accessed May 25. 2022).

Ribaudo, M.O., and R. D. Horan. 1999. The role of education in nonpoint source pollution control policy. *Applied Economic Perspectives and Policy* 21(2): 331–343.

Ribaudo, M., J. D. Kaplan, L. A. Christensen, N. Gollehon, R. Johansson, V. E. Breneman, M. Aillery, J. Agapoff, and M. Peters. 2003. Manure management for water quality costs to animal feeding operations of applying manure nutrients to land. *USDA-ERS Agricultural Economic Report* 824.

Rubæk G. H., K. Kristensen, S. E. Olesen, H. S. Østergaard, and G. Heckrath. 2013. Phosphorus accumulation and spatial distribution in agricultural soils in Denmark. *Geoderma* 209–210: 241–250. https://doi.org/10.1016/j.geoderma.2013.06.022

Schjønning, P., L. W. de Jonge, L. J. Munkholm, P. Moldrup, B. T. Christensen, and J. E. Olesen. 2012. Clay dispersibility and soil friability—Testing the soil clay-to-carbon saturation concept. *Vadose Zone Journal* 11(1).

Soinne, H., J. Hyväluoma, E. Ketoja, and E. Turtola. 2016. Relative importance of organic carbon, land use and moisture conditions for the aggregate stability of post-glacial clay soils. *Soil and Tillage Research* 158: 1-9.

Tisdall, J. M. and J. M. Oades. 1982. Organic matter and water-stable aggregates in soils. *Journal of soil science* 33(2): 141–163.

Turtola E. 1996. Peroxodisulphate digestion and filtration as sources of inaccuracy in determinations of total phosphorus and dissolved orthophosphate phosphorus in water samples containing suspended soil particles. *Boreal Environment Research* 1: 17–26.

van der Salm, C., W. J. Chardon, G. F. Koopmans, J. C. van Middelkoop, and P. A. Ehlert. 2009. Phytoextraction of Phosphorus-Enriched Grassland Soils. *Journal of Environmental Quality* 38: 751–761. https://doi.org/10.2134/jeq2008.0068

Uusi-Kämppä, J., B. Braskerud, H. Jansson, N. Syversen, and R. Uusitalo. 2000. Buffer zones and constructed wetlands as filters for agricultural phosphorus. *Journal of environmental quality* 29(1): 151–158.

Vadas, P. A., P. J. A. Kleinman, A. N. Sharpley, and B. L. Turner. 2005. Relating soil phosphorus to dissolved phosphorus in runoff: A single extraction coefficient for water quality modeling. *Journal of environmental quality* 34(2): 572–580.

Valve, H., P. Ekholm, and S. Luostarinen, 2020. The circular nutrient economy: needs and potentials for nutrient recycling. In *Handbook of the Circular Economy*, ed. M. Brandão, D. Lazarevic, and G. Finnveden, 358–368. Edward Elgar Publishing.

Withers, P.J., P. A. Vadas, R. Uusitalo, K. J. Forber, M. Hart, M., R. H. Foy, A. Delgado, W. Dougherty, H. Lilja, L .L. Burkitt, and G. H. Rubæk. 2019. A global perspective on integrated strategies to manage soil phosphorus status for eutrophication control without limiting land productivity. Journal of environmental quality 48(5): 1234–1246.
